# Connective tissue growth factor stimulates the proliferation, migration and differentiation of lung fibroblasts during paraquat-induced pulmonary fibrosis

**DOI:** 10.3892/mmr.2015.3537

**Published:** 2015-03-24

**Authors:** ZHIZHOU YANG, ZHAORUI SUN, HONGMEI LIU, YI REN, DANBING SHAO, WEI ZHANG, JINFENG LIN, JOY WOLFRAM, FENG WANG, SHINAN NIE

**Affiliations:** 1 Department of Emergency Medicine, Jinling Hospital, Medical School of Nanjing University, Nanjing, Jiangsu 210002, P.R. China; 2 CAS Key Laboratory for Biomedial Effects of Nanomaterials & Nanosafety, National Center for Nanoscience and Technology of China, University of Chinese Academy of Sciences, Beijing 100190, P.R. China; 3 Department of Gastroenterology, The Tenth People’s Hospital of Shanghai, Tongji University, Shanghai 200072, P.R. China

**Keywords:** paraquat, pulmonary fibrosis, connective tissue growth factor, myofibroblast differentiation

## Abstract

It is well established that paraquat (PQ) poisoning can cause severe lung injury during the early stages of exposure, finally leading to irreversible pulmonary fibrosis. Connective tissue growth factor (CTGF) is an essential growth factor that is involved in tissue repair and pulmonary fibrogenesis. In the present study, the role of CTGF was examined in a rat model of pulmonary fibrosis induced by PQ poisoning. Histological examination revealed interstitial edema and extensive cellular thickening of interalveolar septa at the early stages of poisoning. At 2 weeks after PQ administration, lung tissue sections exhibited a marked thickening of the alveolar walls with an accumulation of interstitial cells with a fibroblastic appearance. Masson’s trichrome staining revealed a patchy distribution of collagen deposition, indicating pulmonary fibrogenesis. Western blot analysis and immunohistochemical staining of tissue samples demonstrated that CTGF expression was significantly upregulated in the PQ-treated group. Similarly, PQ treatment of MRC-5 human lung fibroblast cells caused an increase in CTGF in a dose-dependent manner. Furthermore, the addition of CTGF to MRC-5 cells triggered cellular proliferation and migration. In addition, CTGF induced the differentiation of fibroblasts to myofibroblasts, as was evident from increased expression of α-smooth muscle actin (α-SMA) and collagen. These findings demonstrate that PQ causes increased CTGF expression, which triggers proliferation, migration and differentiation of lung fibroblasts. Therefore, CTGF may be important in PQ-induced pulmonary fibrogenesis, rendering this growth factor a potential pharmacological target for reducing lung injury.

## Introduction

Paraquat dichloride (1,1′-dimethyl-4,4′-bipyridinium dichloride; methyl viologen; PQ) is an effective and widely used herbicide that can cause pulmonary fibrosis (1,2). Previous studies have demonstrated that the lungs are one of the primary target organs for PQ-induced toxicity in humans and animals (1,3). The acute toxic effects of PQ, including pulmonary edema and hypoxia, lead to irreversible pulmonary fibrosis. Notably, 1,000s of mortalities due to intentional or accidental ingestion of PQ have been reported (4,5). Although certain drugs, including glucocorticoids, antioxidants and cytotoxic drugs, can mitigate toxicity (6–9), the treatment of acute PQ poisoning is often poor and mortality rates remain high (10,11). Since an effective PQ antidote has yet to be identified, studies on the molecular mechanisms of PQ-induced pulmonary fibrosis are critical for improving treatment and reducing mortality.

Connective tissue growth factor (CTGF) is a cysteine-rich extracellular matrix-associated heparin-binding protein that belongs to the CCN family (12,13). CTGF is widely expressed in numerous tissues at low levels, however, is markedly upregulated in fibrotic and cancerous tissue (14). CTGF regulates various biological processes associated with fibro-genesis, including cellular adhesion, proliferation, migration, differentiation, extracellular matrix (ECM) production (15–17) and angiogenesis (18). In particular, CTGF has been found to promote deposition of several ECM proteins, including collagen, fibronectin and tenascin C (19,20). Aberrant ECM production by lung fibroblasts has been associated with fibrosis in several models of pulmonary injury (21,22). ECM production could further be stimulated as a consequence of lung fibroblast proliferation, migration and differentiation into myofibroblasts (23). Accordingly, CTGF has been demonstrated to affect various cell types involved in the fibrogenic process, including type II alveolar epithelial cells, endothelial cells, mesenchymal stem cells and lung fibroblasts (16).

Although CTGF has been demonstrated to be important in pulmonary fibrosis induced by bleomycin and hyperoxia (24,25), an association between this growth factor and PQ-induced lung injury has not been established. In particular, the effect of CTGF on ECM production, cellular proliferation, migration and myofibroblast differentiation in association with PQ poisoning remains to be elucidated. The aim of the present study was to investigate the effects of PQ on CTGF expression and subsequent pulmonary fibrosis.

## Materials and methods

### Ethics statement

All animals were handled in accordance with guidelines approved by the Experimentation Ethics Review Committee of Nanjing University (Nanjing, China). The rats were fed with commercial rat chow, provided with water *ad libitum* and kept on a 12:12 h light-dark cycle.

### Cell culture

MRC-5 lung fibroblasts (human lung fibroblasts; American Type Culture Collection, Manassas, VA, USA; cat. no. CCL 171) were cultured in high Dulbecco’s modified Eagle’s medium (DMEM; HyClone Laboratories, Inc., Logan, UT, USA) with 10% fetal bovine serum (FBS; Invitrogen Life Technologies, Carlsbad, CA, USA) supplemented with 1% L-glutamine and 1% penicillin/streptomycin solution. Cells were incubated at 37°C in 5% CO_2_ and routinely passaged upon reaching 80% confluency, using 0.25% trypsin and a 1:3 cell dilution for each passage.

### Cell viability

The viability of lung fibroblasts was evaluated using a Cell Counting kit-8 (CCK-8; Dojindo Laboratories, Kumamoto, Japan) assay. Cells were plated in 6-well plates at a density of 2×10^6^ cells/ml for 12 h and treated with various concentrations of CTGF (50–200 ng/ml; PeproTech, Inc., Rocky Hill, NJ, USA) for 24, 48 or 72 h. The cells were then transferred into a 96-well culture plate (n=8) at a density of 2×10^4^ cells/100 *μ*l/well for 12 h. The culture medium was removed and 100 *μ*l of serum-free medium containing 10 *μ*l of CCK-8 solution was added to each well. After a 4 h incubation period, the absorbance was measured at an optical density (OD) of 450 nm using a multi-detection microplate reader (VersaMax; Molecular Devices, Sunnyvale, CA, USA).

### Cell migration

A double-chamber system was used to perform a transwell migration assay to determine the migration ability of lung fibroblasts. Cells were seeded in a 6-well plate with serum-free medium at a density of 4×10^4^ cells/per upper chamber. Culture medium (1 ml) with 10% FBS containing various concentrations of CTGF (50–200 ng/ml) was simultaneously added to the lower chamber. The upper and lower chambers were separated by a permeable polycarbonate membrane with a pore size of 8 *μ*m. The cells were permitted to migrate to the lower chamber for 24 h. Following this incubation period, cells that had entered the lower surface of the filter membrane were fixed with a cold solution of 4% paraformaldehyde for 30 min. Cells were then washed three times with phosphate-buffered saline (PBS) and stained with 0.1% crystal violet for 30 min. Cells that remained in the upper surface were gently scraped off with a cotton swab. A total of 10 different fields of view from each membrane were randomly selected and captured using a photomicroscope (BX51; Olympus, Tokyo, Japan). Cell migration was quantified by counting the number of migrated cells. Experiments were performed three times in duplicate.

### Animal groups and experimental protocol

A total of 48 adult male Sprague-Dawley (SD) rats weighing 200–250 g were purchased from the Animal Center of Nanjing University (Nanjing, China). The SD rats were randomly divided into two groups (n=24 for each group). The experimental group received a single intraperitoneal injection of PQ (30 mg/kg), while the control group received an equivalent volume of sterile saline. The rats were then sacrificed by cervical dislocation after 7, 14 and 28 days.

### Histological analysis

Following sacrificing the animals, the lungs were removed and the left lung was fixed with 4% paraformaldehyde for 16 h. The tissue samples were then processed using graded alcohol, xylene and paraffin and blocked in paraffin. The paraffin-embedded sections (5 *μ*m thick) were stained using a hematoxylin and eosin (H&E) kit (Biyuntian, Inc., Nantong, China) and a Masson’s trichrome staining kit (Nanjing Jiancheng Bioengineering Institute, Nanjing, China), according to the manufacturer’s instructions. The slides with H&E staining were examined using light microscopy (Eclipse TE2000-S; Nikon Corporation, Tokyo, Japan) and images were captured to determine the integrity of the tissue. The severity of pulmonary fibrosis in the lung sections stained for collagen with Masson’s trichrome stain was determined by a histopathologist who was blinded to the protocol design.

### Immunohistochemical staining

Formalin-fixed, paraffin-embedded skin sections were stained with rabbit polyclonal anti-CTGF antibodies (cat. no. ab6992; Abcam, Inc., Cambridge, MA, USA). Following transferal through a graded series of alcohol and xylene, lung tissue samples were embedded in paraffin and sectioned (5 *μ*m). The sample sections were mounted onto poly-L-lysine-coated slides and processed for immunohistochemical analysis, according to a similar procedure as previously reported (26). Briefly, sections were incubated overnight at room temperature with 3% bovine serum albumin (BSA) for 30 min at 37°C for blockade of non-specific binding sites and then incubated overnight at 4°C with the primary antibody targeting CTGF (1:200 dilution). Specificity of the antibody was examined using normal rabbit serum instead of the primary antibody. The slides were then incubated with a secondary biotinylated goat anti-rabbit antibody (1:200 dilution; cat. no. BA1003; Wuhan Boster Biological Technology, Ltd., Wuhan, China) for 30 min at 37°C. Following rinsing, the slides were incubated with horseradish peroxidase-conjugated streptavidin and then washed with deionized water. The samples were then exposed to 3,3′-diaminobenzidine substrate solution for 10 min, coun-terstained with hematoxylin and mounted with coverslips. Images were captured on a Nikon Eclipse TE2000-S microscope (Nikon Corporation). A brown reaction product was considered a positive result.

### Immunofluorescence

To evaluate the effect of CTGF on myofibroblast differentiation of human lung fibroblasts, MRC-5 lung fibroblasts were treated with 100 ng/ml CTGF (PeproTech, Inc.) for 3 days. Immunofluorescence staining was performed as previously described (21). Briefly, MRC-5 cells were fixed with 4% paraformaldehyde and permeabilized with 0.1% Triton X-100 for 10 min. The cells were then incubated with PBS containing 2% BSA for 1 h at 37°C to block unspecific binding sites. The fixed cells were then incubated with the following primary antibodies: Rabbit polyclonal anti-α-smooth muscle actin (α-SMA; 1:200 dilution; cat. no. ab5694) and rabbit polyclonal anti-collagen I (1:200 dilution; cat. no. ab34710; Abcam, Inc., Cambridge, MA, USA) at 4°C for 16 h. Following three washes with PBS, cells were incubated with a secondary antibody (1:400 dilution; goat anti-rabbit Alexa Fluor 594 or 488; Invitrogen Life Science, Gaithersburg, MD, USA) in 2% BSA for 1 h at 37°C in the dark. Nuclear staining was performed using 5 mg/ml 4′,6-diamidino-2-phenylindole (Biyuntian, Inc.). Cells were visualized using a confocal fluorescence microscope (Fluoview FV10i; Olympus).

### Western blot analysis

In order to investigate the effects of PQ on protein levels of CTGF, MRC-5 cells were treated with various concentrations of PQ (50–500 *μ*M; Sigma-Aldrich, St. Louis, MO, USA) for 72 h. Protein samples were obtained from cultured cells and animals (right lung, n=8) treated with PQ. Western blot analysis of protein lysates was performed as previously described (21). Briefly, cells and lung tissues were lysed in ice-cold RIPA extraction buffer (150 mM NaCl, 10 mM Tris-HCl, pH 7.4, 1% Triton X-100, 1% sodium deoxycholate and 0.1% SDS) containing a protease inhibitor cocktail (Roche Diagnostics, Indianapolis, IN, USA) for 30 min. The whole lysates were then centrifuged at 12,000 × g for 30 min and the protein concentration in the supernatant was determined using a bicinchoninic acid assay (Wuhan Boster Biological Technology, Ltd.). The protein samples were boiled for 10 min and 20 *μ*l aliquots were then subjected to 12% SDS-polyacrylamide gel electrophoresis. The protein bands were electrophoretically transferred onto a polyvinylidene fluoride membrane, which was incubated in blocking buffer (1X PBS, 0.1% Tween-20, 1% BSA and 5% non-fat milk) for 1 h at 37°C. The membrane was then exposed to primary antibodies (at a 1:3,000 dilution) against rabbit α-SMA, rabbit collagen I and mouse β-actin (Abcam, Inc.) in blocking buffer overnight at 4°C. Following three washes in PBS containing 0.05% Tween 20, the membrane was incubated with the secondary antibody (horseradish peroxidase-conjugated goat anti-rabbit/mouse IgG; Wuhan Boster Biological Technology, Ltd.) at 37°C for 1 h. Visualization of immunoreactive protein bands was performed with an enhanced chemiluminescence detection kit (Amersham Biosciences, Piscataway, NJ, USA) using an Odyssey Scanning System (LI-COR, Inc., Lincoln, NE, USA).

### Hydroxyproline (HP) content

The quantity of collagen in the lung tissue was determined by analysis of HP content according to the manufacturer’s instructions of the detection kit (Nanjing Jiancheng Bioengineering Institute). The absorbance was measured at 550 nm and the HP content was determined using a standard curve (0–100 mg/ml).

### Statistical analysis

Data are expressed as the mean ± standard deviation. Differences among groups were evaluated by one-way analysis of variance using Statistical Package for the Social Sciences (SPSS) version 18.0 software (SPSS, Inc., Chicago IL, USA). P<0.05 was considered to indicate a statistically significant difference.

## Results

### PQ-induced lung injury and pulmonary fibrosis

**Figure 1:**
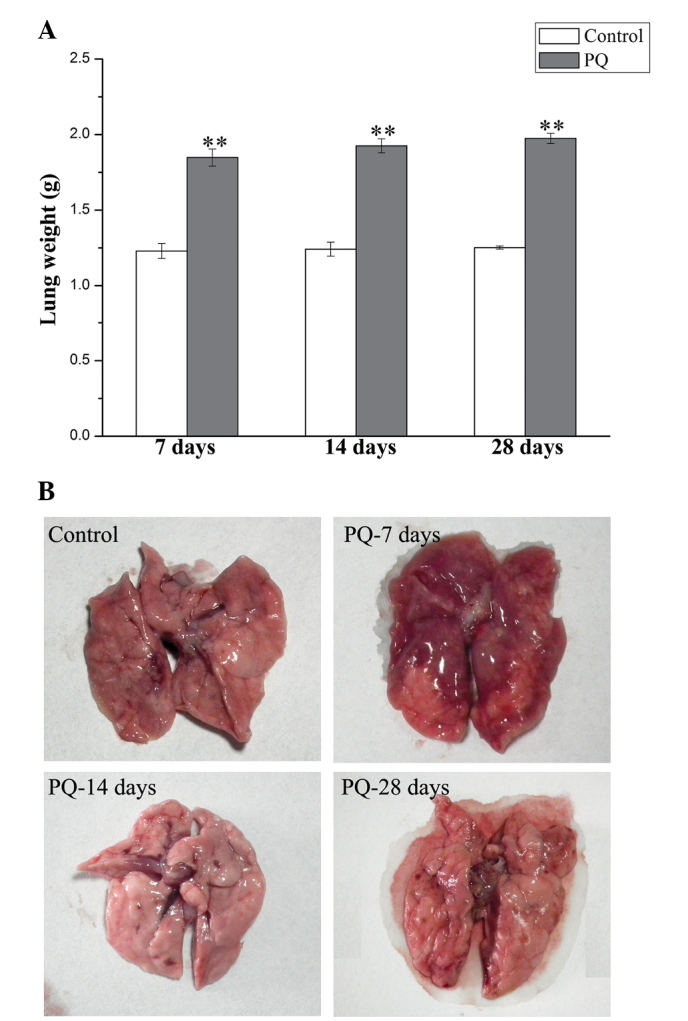
PQ-induced lung injury and pulmonary fibrosis. (A) Weights of lung tissue in the control and PQ-treated mice 7, 14 and 28 days after PQ administration. Data are expressed as the mean ± standard deviation. ^**^P<0.01 vs. the control group (n=8). (B) Representative images of whole lungs 7, 14 and 28 days after PQ administration. PQ, paraquat.

After PQ administration for 7, 14 and 28 days, rats were sacrificed and the lungs were collected. As shown in Fig. 1A, the weight of the lungs increased in response to PQ administration, indicating the presence of pulmonary inflammation and fibrosis. In addition, the macroscopic appearance of the lungs was fibrotic (Fig. 1B). These results suggest that PQ is able to induce lung injury, inflammation and fibrosis in this rat model.

### Histopathological analyses of rat lungs treated with PQ

**Figure 2:**
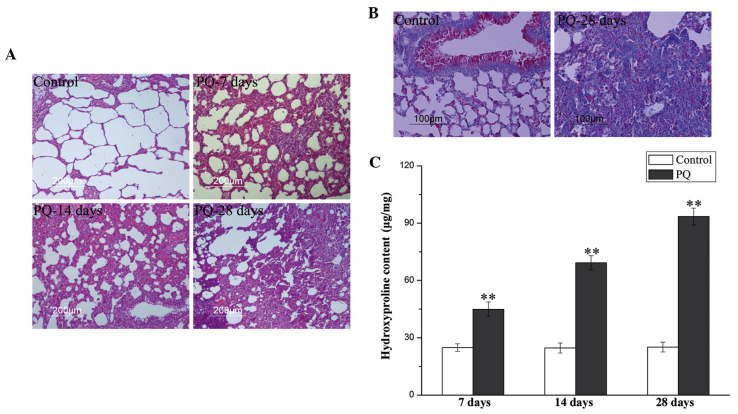
PQ-induced pulmonary fibrogenesis and collagen deposition in rat lung tissue. (A) Histopathological views of lung tissues from rats following PQ exposure (7, 14 and 28 days). Lung sections were stained with hematoxylin and eosin. (B) Masson’s trichrome staining analysis of collagen (blue color) on day 28 following PQ exposure. (C) Hydroxyproline content in rat lungs on day 7, 14 and 28 following PQ administration. All data are presented as the mean ± standard deviation. ^**^P<0.01 vs. the control group (n=8). PQ, paraquat.

To investigate the effects of PQ on lung tissue, serial lung sections from PQ-treated rats (7, 14 and 28 days after PQ administration) were stained with H&E or Masson’s trichrome and examined using a light microscope. Representative histological sections from lungs of rats in each experimental group are shown in Fig. 2A. Lung tissues in the control group appeared histologically normal, showing no signs of inflammation or epithelial damage. By contrast, histopathological examination of the lung tissue following PQ administration revealed alterations in the lung structure. After 7 days, the lungs exhibited marked alterations in tissue structure, exhibiting signs of acute injury with interstitial edema and widespread inflammatory cell infiltration in the alveolar space and septum. In addition, an infiltration of mononuclear inflammatory cells, fibroblast proliferation, extensive cellular thickening and fibrosis was evident after 14 days. Similarly, at day 28, H&E staining indicated thickened alveolar walls and pulmonary interstitial fibrosis. Masson’s trichrome staining revealed that collagen deposition (blue staining) in the PQ group was increased compared with the control group (Fig. 2B). In conclusion, histological examination demonstrated that PQ administration induced lung injury and pulmonary fibrosis.

### HP content in the lungs following PQ administration

In order to evaluate collagen deposition in the lung tissues, the HP content was determined 7, 14 and 28 days after PQ administration. As shown in Fig. 2C, PQ caused a statistically significant (P<0.05) increase in the amount of HP at all time points. On day 28, the HP content in the PQ group was more than three times higher than in the control group. The results of HP quantification were consistent with the histopathological results.

### PQ induces expression of CTGF in lung tissues and lung fibroblasts

**Figure 3:**
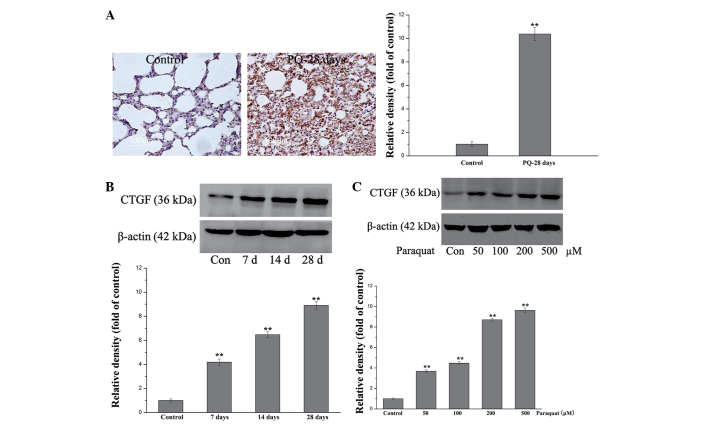
PQ-induced CTGF expression in rat lungs and MRC-5 human lung fibroblast cells. (A) Immunohistochemistry of CTGF in lung tissue 28 days after PQ administration. (B) Western blot analysis of CTGF in lung tissues 7, 14 and 28 days after PQ exposure (P<0.01). (C) Western blot analysis of CTGF in MRC-5 cells exposed to 50, 100, 200 and 500 *μ*M of PQ. Western blot bands were quantified using densitometry analysis and normalized to the expression of β-actin. Densitometry data are presented as the mean ± standard deviation. ^**^P<0.01 vs. the control group (n=6) for each experiment. PQ, paraquat; CTGF, connective tissue growth factor.

Immunohistochemical staining was used to detect the expression of CTGF in lung tissue. As shown in Fig. 3A, the CTGF expression was significantly increased 28 days after PQ administration (P<0.01). Western blot analysis was also performed to confirm that the CTGF expression was enhanced in response to PQ. As shown in Fig. 3B, PQ-induced a time-dependent increase in CTGF protein levels. Furthermore, western blot analysis was performed using MRC-5 cell lysates to evaluate whether the same trend could be observed *in vitro*. Following exposure to various concentrations of PQ (50–500 *μ*M), the results indicated that the cells exhibited significantly higher levels of CTGF protein (P<0.01; Fig. 3C). The results suggest that PQ-induced CTGF expression may contribute to pulmonary fibrogenesis.

### CTGF promotes the proliferation and migration of lung fibroblasts

**Figure 4:**
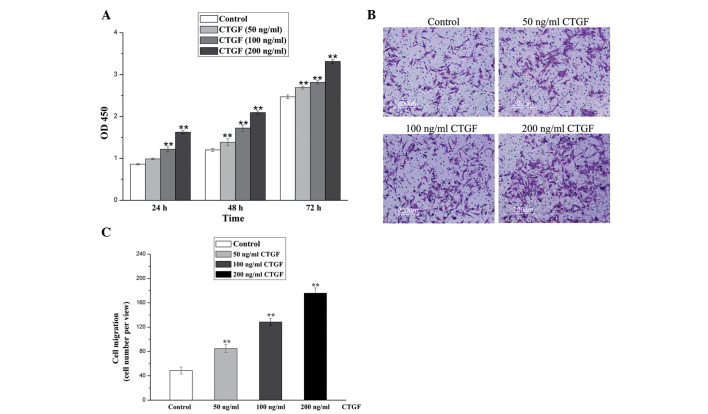
CTGF-induced proliferation and migration of lung fibroblasts. (A) Cell proliferation of MRC-5 cells exposed to 50, 100 and 200 ng/ml of CTGF (n=6). (B) Transwell migration analysis of MRC-5 cells treated with 50, 100 and 200 ng/ml of CTGF. The images are representative of three independent experiments. (C) Quantitative presentation of cell migration ability. The data are presented as the mean ± standard deviation from three experiments. ^**^P<0.01, vs. the control group. PQ, paraquat; CTGF, connective tissue growth factor.

The effect of CTGF on the proliferation and migration of lung fibroblasts was evaluated, as these biological processes are important in pulmonary fibrogenesis. As shown in Fig. 4A, the cell viability was significantly increased in response to CTGF (P<0.01). The data indicate that CTGF promotes cell proliferation in a dose- and time-dependent manner. As shown in Figs. 4B and C, the results from the transwell migration assay reveal that CTGF improves lung fibroblast migration in a statistically significant manner (P<0.01). In particular, 200 ng/ml of CTGF caused a 3.6-fold increase in cell migratory capacity. These results highlight the possibility that CTGF is able to mediate PQ-induced pulmonary fibrogenesis.

### CTGF induces myofibroblast differentiation of lung fibroblasts

**Figure 5:**
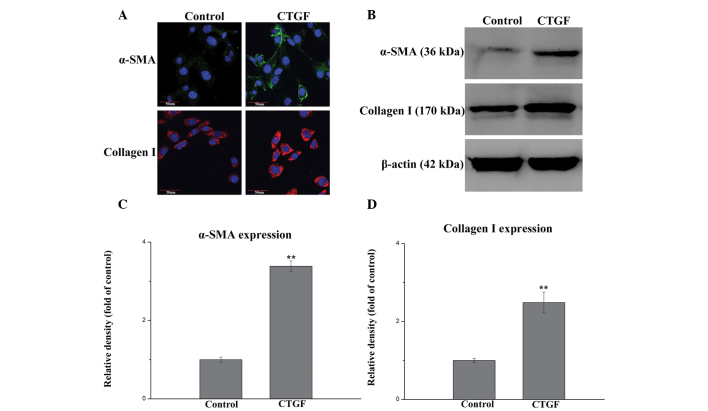
CTGF-induced differentiation of MRC-5 lung fibroblasts into myofibroblasts. (A) Immunofluorescence analysis of α-SMA and collagen I in MRC-5 fibroblasts following treatment with CTGF for 3 days. Magnification, ×600 (scale bar=50 *μ*m). (B) Western blot analysis of α-SMA and collagen I in whole cell lysates. β-actin was used as a loading control. Quantitative analysis of western blot results of (C) CTGF-induced α-SMA expression and (D) collagen I expression. Protein bands were quantified by densitometry and normalized to the expression of β-actin. Densitometry data are presented as the mean ± standard deviation. ^**^P<0.01 vs. the control group. n=6 for each group. α-SMA, α-smooth muscle actin; CTGF, connective tissue growth factor.

To further investigate the role of CTGF in pulmonary fibrogenesis, the differentiation of lung fibroblasts into myofibroblasts was examined. MRC-5 cells were incubated with 100 ng/ml of CTGF for 3 days. Immunofluorescence and western blot analysis were used to measure expression levels of α-SMA and collagen I, which are myofibrolast differentiation markers. As shown in Fig. 5A, immunofluorescence staining indicated that α-SMA (stained green) and collagen I (stained red) protein expression was increased in the CTGF-induced group, as compared with the control group. In addition, as shown in Figs. 5B–D, western blotting demonstrated that CTGF induced a pronounced increase in the protein expression levels of α-SMA and collagen I, indicating the differentiation of lung fibroblasts into myofibroblasts. This differentiation process has previously been associated with lung fibrosis (27).

## Discussion

PQ has previously been found to cause acute lung injury and pulmonary fibrosis with interstitial collagen deposition, which leads to reduced functional capacity (1). PQ poisoning is a severe health problem, as numerous human mortalities have occurred as a consequence of PQ ingestion (4,5). The lung is the major target organ for this toxic agent, as alveoli type II epithelial cells absorb PQ through an active polyamine uptake process (28–30). PQ can accumulate in lung tissue and reach peak plasma concentrations within 2 h after ingestion (31). Notably, the concentration of PQ in the lung parenchyma can be 10–20 times higher than that in the plasma (32). The signaling pathways that lead to PQ-induced pulmonary fibrosis remain to be elucidated. Previous studies have focused on clarifying the molecular mechanisms of PQ poisoning to determine useful molecular targets for developing therapeutic strategies. The present study examined the role of CTGF in PQ-induced collagen production and myofibroblast differentiation of human lung fibroblasts.

CTGF is a downstream cooperative mediator of the transforming growth factor-β signaling pathway and is widely expressed in numerous tissues at low physiological levels. However, this growth factor is markedly upregulated at the pathological sites of numerous animal models of human disease, including pulmonary fibrosis, liver fibrosis, skin fibrosis, cancer and various types of malignancy (14,33,34). In particular, increased levels of CTGF have previously been reported in patients with severe pulmonary fibrosis and animal models of pulmonary fibrosis (25). In the present study, PQ exposure caused alterations in lung architecture, which was evident from interstitial edema, extensive cellular thickening of interalveolar septa, increased interstitial cells with a fibroblastic appearance and excessive collagen deposition. Concurrently, it was found that PQ exposure induces CTGF expression *in vitro* and *in vivo*. A previous *in vitro* study also indicated that CTGF can exert an effect on a number of cell types, thereby promoting biological processes associated with fibrogenesis, including cell proliferation, migration and ECM production (16). The present study demonstrated that CTGF can induce the proliferation and migration of MRC-5 lung fibroblasts. Therefore, it is likely that CTGF is a mediator of PQ-induced pulmonary fibrogenesis.

Accumulating evidence suggests that the overexpression of collagen and α-SMA in lung fibrotic lesions is associated with fibrotic lung disease (35). Collagen is the major ECM component of the lungs and increases at the early stages of acute lung injury, affecting respiratory mechanics. Myofibroblasts that originate from the differentiation of lung fibroblasts exhibit morphological and biochemical characteristics of lung fibroblasts and smooth muscle cells, which have been considered the main source of ECM within the impaired lungs of patients with idiopathic pulmonary fibrosis (36,37). The results from the present study are supported by a previous study demonstrating that CTGF knockdown animals have a reduced pathogenic fibrotic response (29). The mechanism responsible for protection against fibrosis in CTGF knockdown cells was found to be reduced collagen synthesis (38). In the present study, the results indicate that PQ exposure causes increased levels of collagen and CTGF, in a time-dependent manner. Immunofluorescent staining and western blot analysis reveal that lung fibroblasts treated with CTGF have increased expression of collagen and α-SMA. This observation suggests that CTGF can induce myofibroblast differentiation.

In conclusion, our observations imply that PQ-induced overexpression of CTGF may be responsible for pulmonary fibrosis, through promoting the proliferation, migration and myofibroblast differentiation of lung fibroblasts. The present study supports the theory that pharmacological inhibition of CTGF is a feasible strategy to reduce the magnitude of pulmonary fibrosis induced by PQ.
